# Incorporation of a dual-task cognitive exercise during unsupported seated balance in collegiate para athletes

**DOI:** 10.3389/fcogn.2026.1826820

**Published:** 2026-06-22

**Authors:** Ryan N. Moran, Alexandra C. Curry

**Affiliations:** Athletic Training Research Laboratory, Department of Health Science, The University of Alabama, Tuscaloosa, AL, United States

**Keywords:** balance, cognition, disability, postural control, sports medicine

## Abstract

**Introduction:**

Balance remains a critical element in the evaluation and management of sports injury, with recent recommendations to integrate dual-task cognitive exercises into balancing to further challenge the body's vestibular and somatosensory systems. Dual-task balancing has yet to be examined in a sample of para-athletes. The purpose of this study was to examine the effects of a dual-task cognitive exercise on unsupported, seated balance in a sample of healthy, collegiate para-athletes. A secondary objective was to investigate the role of sex, concussion history, and functional classification.

**Methods:**

A total of 52 participants completed a seated balance battery, with their eyes open and closed, on a stable and unstable surface, under both single and dual-task conditions. Measures consisted of human-scored biomechanical errors of coming out of the seated positions.

**Results:**

Between 90 and 94% of participants committed 0 errors during stable and unstable single-task, along with stable dual-task, while only 86.5% had 0 errors for unstable, dual-task balancing. No sex differences were observed during single and dual-task balancing. Concussion history and functional classification did not affect performance.

**Discussion:**

The lack of differences between single and dual-task indicates that dual-task cognitive exercises or human-scored errors may not appropriately affect and assess upright seated balance and may require more dynamic tasks to further challenge balance and postural stability.

## Introduction

Upright, seated balance is a critical element for wheelchair users for transfers and improving core stability and posture. Balance also remains a key component of sports injury evaluation and rehabilitation, often associated with lower extremity musculoskeletal pathologies (Zheng et al., 2025), neurological conditions (Handiru et al., 2024; Joyce et al., 2022; Korkusuz et al., 2026; Hida et al., 2025), and head injury (Murray et al., 2014). Balancing, while routinely administered with patients standing either on one or both feet, evaluates the central nervous system, and requires control from the vestibular, visual, and somatosensory systems. A variety of clinical balance assessments, such as the balance error scoring system (BESS) (Ozinga et al., 2018), Romberg test (Mermelstein et al., 2026), and the modified clinical test of sensory interaction in balance (mCTSIB) (Moran et al., 2020a), are used to recognize potential injury via inability to complete the tasks or excessive sway and also to correct central nervous system impairments and prevent future risk of falling.

The Sport Concussion Assessment (SCAT6) (Echemendia et al., 2023) and Office (SCOAT6) (Patricios et al., 2023) Assessment Tools incorporate multifaceted balance assessment, consisting of the static, standing balance and multiple variations of tandem gait (e.g., heel-to-toe walking). A newly incorporated item in the SCAT6 and SCOAT6, a change from its prior iteration, is the option for a dual-task condition during tandem gait. Dual-task cognitive exercises are widely used clinically as a means to further challenge balance by having to handle multiple demands (Howell et al., 2018), such as counting or spelling backwards, while maintaining upright, postural or motor control. Dual-task balance typically presents clinically with increased postural sway and increased completion time for timed tandem gait (Buttner et al., 2020; Howell et al., 2020, 2013; Oldham et al., 2021).

When considering balance assessments for wheelchair-using para-athletes, tandem gait tasks are unable to be completed due to the heel-to-toe walking nature of the tasks. Additionally, the BESS and it's modified version (mBESS) are a challenging due to inability to stand on one and both feet (Weiler et al., 2021; Smetana et al., 2024). Researchers developed the Wheelchair Error Scoring System (WESS) (Moran et al., 2020b) as a similar clinical tool to the mBESS, which consists of seated, upright balancing out of a wheelchair and maintaining a wheelie (e.g., lifting front wheels of wheelchair off the ground) for 20-s each. Moran et al. (2020b) examined preliminary, baseline WESS performance for collegiate para athletes, revealing that few biomechanical errors are made by this sample, especially in the upright, seated tasks out of the wheelchair, despite using both a firm and unstable surface. While these upright, seated tasks are also relevant to patient transfers from and to a wheelchair, these conditions can be utilized clinically for both the assessment and management of neurological injury, whether existing pathologies, such as spinal cord injury (SCI) and spina bifida, but also sport-related concussion. Therefore, it is worth exploring the effects of dual-task conditions of seated, upright balance in this sample.

The purpose of this project was to examine the effects of a dual-task cognitive exercise on unsupported, seated balance in a sample of healthy, collegiate para-athletes. A secondary objective was to investigate the role of sex, concussion history, and functional classification. It was hypothesized that dual-task conditions would result in worse balance than single-task conditions. Additionally, we hypothesized that no differences would exist during single and dual-task conditions between sexes or concussion history, while lower functional classifications would have greater dual-task deficits compared to higher classifications.

## Materials and methods

### Participants

A total of 52 collegiate para-athletes, aged 18 to 28 years (21.19 ± 2.6) from a national sample of men's and women's wheelchair basketball participated in this study. Regarding demographics, there was even distribution between sexes (*n* = 26, 50.0%), 23 (44.2%) participants reported a history of concussion, while the majority of functional classifications ranged from 1.0 (15.4%) to 3.0 (15.4%). The specific injury or pathology of the athletes varied widely, with 23 unique diagnoses. The most common diagnoses were spina bifida (38.5%, *n* = 20) and SCI (17.3%, *n* = 9). Full demographics are provided in Table 1.

**Table 1:** Demographics of the sample.

Age in years	
Mean ± SD	21.19 ± 2.6
Male only	21.58 ± 2.5
Female only	20.81 ± 2.6
Concussion history	
No history	55.8 (29)
Prior history	44.2 (23)
1 prior concussion	43.5 (10)
2 prior concussions	47.8 (11)
3+ prior concussions	8.7 (2)
Functional classification	
1.0—no active trunk movement	15.4 (8)
1.5	21.2 (11)
2.0—active upper trunk movement	15.4 (8)
2.5	15.4 (8)
3.0—active upper and lower trunk movement	15.4 (8)
3.5	7.7 (4)
4.0—maximal trunk movement with weakness	5.8 (3)
4.5—maximal trunk movement with no weakness	3.8 (2)

All demographics provided are % (*n*) unless otherwise specified.

### Procedures

Participants arrived at the athletic training facility of the institution during a designated research time, prior to any physical activity or conditioning of that day, to negate any exercise effects or fatigue. The balance battery consisted of being seated upright and out of their wheelchair on a height-adjustable table in two different conditions: (i) seated on a firm surface with eyes closed, (ii) seated on an unstable surface with eyes closed. The unstable surface consisted of the participant sitting on a 6 cm-thick, polyvinyl chloride foam AirEx balance pad (Stanek et al., 2013), which is widely used in sports medicine and rehabilitation. The two conditions were first completed under single-task, which required the athlete to focus solely on upright, balance. Then the conditions were repeated under dual-task conditions, which consisted of serial subtraction by 4s, 6s, or 7s from a randomly presented 2- or 3-digit number (Moran et al., 2025). Single task conditions were completed first for alignment with clinical balance tool recommendations. All conditions were timed for 20-s each, for consistency with the WESS (Moran et al., 2020b) and BESS (Ozinga et al., 2018; Moran et al., 2025). Measures consisted of human-rated errors of the WESS, as reported by Moran et al. (2020b) with the exclusion of “coming out of the wheelie” and “changing grip during the wheelie task” as the wheelie task was eliminated in this study. Errors were scored from 0 (none) to 10 (maximum) per condition with more errors indicating worse balance and postural control during each task. All scoring was completed by the same individual to control for interrater differences and agreement.

### Statistical analyses

General descriptive (i.e., means, SDs, frequencies) and inferential statistics were used to summarize all demographic information and balance errors. Due to non-parametric data, medians for all balance errors were 0. Means and SDs are provided for ease of clinical utility. Wilcoxon signed-rank tests were conducted to determine differences in errors between single and dual-task conditions. A series of Mann-Whitney *U* tests were conducted to determine differences between modifier groups of sex (male and female) and concussion history (prior history or no history). A Kruskal–Wallis *H* test was used for group differences by functional classification (1.0–1.5, 2.0–2.5, and 3.0–4.5).

## Results

### Baseline and dual-task performance

Regarding balance errors, 86.5% (*n* = 45) committed 0 errors, with 9.6% (*n* = 5) and 13.4% (*n* = 7) committing errors across single and dual-task conditions, respectively (Table 2). There was no significant difference in errors from single to dual-task during the firm (*p* = 0.31) and unstable (*p* = 0.06) surface conditions. Of the two participants that made errors on single-task firm surface, only one saw an increase of errors during dual-task, with one extra error. On the unstable surface, only four participants had an increase in errors from single to dual-task.

**Table 2:** Balance performance for the sample.

Balance condition	Mean ±SD	Range (min–max)	% (*n*) Zero scoring
Firm surface—single task	0.08 ± 0.4	3 (0–3)	94.2 (49)
Foam surface—single task	0.15 ± 0.6	4 (0–4)	90.3 (47)
Firm surface—dual task	0.10 ± 0.5	4 (0–4)	94.2 (49)
Foam surface—dual task	0.27 ± 0.8	4 (0–4)	86.5 (45)

Zero scoring indicates 0 errors on balance battery.

### Sex, concussion history, and functional classification effects

Sex differences were not apparent across single-task firm (*U* = 337.5, *p* = 0.97) and unstable (*U* = 324.0, *p* = 0.61) surfaces, nor dual-task firm (*U* = 337.5, *p* = 0.97) and unstable (*U* = 328.0, *p* = 0.75) surfaces (Table 3). Additionally, no differences were observed between concussion history groups and errors (*U* range = 281.0–310.0, *p* range = 0.10–0.25). Regarding functional classification, no differences existed across balance conditions (*H* range = 1.2–0.04, *p* range = 0.54–0.97).

**Table 3:** Balance performance by modifier.

Conditions	Sex			Concussion history (Hx)			Functional classification			*p*
	Male	Female	*p*	Prior Hx	No Hx	*p*	1.0 and 1.5	2.0 and 2.5	3.0—4.5	
Firm—Single	0.04 ± 0.2	0.12 ± 0.5	0.97	0.00 ± 0.0	0.13 ± 0.6	0.20	0.00 ± 0.0	0.06 ± 0.2	0.18 ± 0.7	0.54
Unstable—Single	0.08 ± 0.2	0.23 ± 0.8	0.61	0.04 ± 0.2	0.24 ± 0.8	0.25	0.15 ± 0.4	0.13 ± 0.3	0.29 ± 1.0	0.97
Firm—Dual	0.04 ± 0.2	0.15 ±0.7	0.97	0.00 ± 0.0	0.17 ± 0.7	0.20	0.00 ± 0.0	0.06 ± 0.2	0.24 ± 0.9	0.54
Unstable—Dual	0.23 ± 0.6	0.31 ±0.9	0.79	0.13 ± 0.6	0.38 ± 0.9	0.10	0.30 ± 0.6	0.13 ± 0.3	0.41 ± 1.1	0.77

## Discussion

The findings of this study indicated that the incorporation of a dual-task condition during upright, seated balance did not lead to increased errors, as hypothesized. While dual-task conditions have been widely established to lead to increases in tandem gait time and worse postural control (Oldham et al., 2021; Howell et al., 2017), our findings are consistent with Moran et al. (2025) that did not observe dual-task differences in the mBESS. Our findings help to further support the notion that dual-task worsening may be from more dynamic motor tasks (e.g., tandem gait or wheelchair propulsion) where both the mBESS and our study's balance conditions were static in nature, having to remain upright postural control. Salm et al. (2025) noted that a dual-task condition resulted in higher wheelchair propulsion frequency, shorter cycle, and lower propulsion angle in middle aged wheelchair users, indicating a spatiotemporal influence from dual-task. It should be noted that the participants in our study were seated, rather than completing a dynamic propulsion task. For this study, we only considered the use of biomechanical errors for clinical purposes and not a combined use of force-platform or motion capture technology. However, Moran et al. (2025) noted both a lack of single to dual-task differences of the mBESS, indicating that the relationship between errors and postural sway may be strongly correlated. Thus, any increase in errors from single to dual-task would have been associated with more objective increased in postural sway (Moran and Haller, 2024). It is important to note, given the high prevalence of error-free scoring at baseline, caution is needed, as there may be a potential ceiling effect that that task may not be challenging enough, causing the tests ability to distinguish different levels of balance ability, unlike the mBESS using challenging stances, such as single-leg and tandem stances.

A secondary purpose of this study was to examine the role of sex, concussion history, and functional classification on our balance battery. Across all three variables, no differences were observed between groups, indicating stability for all three modifiers. The lack of differences between sex may be justified by prior research on the WESS, yielding no differences in eyes open and eyes closed seated balance on a firm surface, balance disk conditions, and a wheelie task (Moran et al., 2020b). Sex differences in balance have largely been attributed to lower centers of gravity (Moran et al., 2020a), which may be negated in a wheelchair population. Additionally, recent research has noted a lack of sex differences on the mBESS across competition levels of high school, club sport, and collegiate athletes (Moran and Haller, 2024), so it is possible that sex remains a non-modifier among more static balance tests. Our lack of differences in concussion history may also be attributed to the balance task at hand, in that the upright seated conditions do not stress the central nervous system or motor trunk control, similarly to sex. Harper et al. (2021) noted that WESS performance was not altered by concussion history or qualifying disability, which further support our findings on concussion history and functional classification. Findings in a similar study of collegiate para-athletes noted that functional classification affected baseline Vestibular/Ocular Motor Screening with greater symptom provocation during saccadic eye movements, gaze stability, and visual motion sensitivity in higher classifications (Moran et al., 2024). However it should be noted that these items involve eye and head movements, whereas our seated, upright task involved the head and eyes remaining still, which may also contribute to the lack of group differences.

Since the 6th International Consensus Statement on Concussion in Sport (Patricios J. S. et al., 2023) and the Concussion in Para Sport Group's Position Statement (Weiler et al., 2021), para athletes have been receiving growing consideration in the sports medicine landscape. Recent initiatives look to further advance concussion assessment and clinical care for para sport athletes, which aim to validate new balance assessments specific to this population, with clinical translation and similarities to non-para-athletes (Post et al., 2026). This study was not without limitations. First, we used a national sample of para-athletes that use a wheelchair for daily living. It is possible that dual-task differences may be apparent in amputee athletes or other spinal pathologies, that are able to complete more objective, laboratory grade assessments. Additionally, we did not utilize force-platform technology that may be more sensitive to subtle changes in postural control during the upright, seated task, as well as center of pressure changes occurring in the absence of biomechanic, human-scored errors. Lastly, all single-task assessments were completed prior to dual-task conditions for consistency with clinical tool recommendations, which may yield different findings if counterbalanced, due to learning effects or fatigue. We did not record the mathematical/cognitive errors, which can better understand dual-task cost and task-prioritization. Future research should consider utilizing this technology and motion capture data to measure neurological changes during wheelchair transfers, rather than seated, upright balance. Additionally, future research should consider these measures post-concussion and aim to quantify specific errors, if using human-scored errors for clinical utility.

In conclusion, the incorporation of a dual-task condition did not worsen balance, as measured by human-scored errors. This is clinically useful in knowing that dual-task exercises may not yield the similar results that we see in dynamic motor tasks in non-para-athletes. When rehabilitating the para-athlete for balance deficits, clinicians should consider more difficult and challenging conditions or parameters such as incorporating perturbations, head movements, and upper extremity tasks while balancing. Additionally, modifiers of concussion, such as sex and concussion history did not differ on balance which shows stability across groups.

## Data Availability

The datasets presented in this article are not readily available because of institutional policy. Requests to access the datasets should be directed to rnmoran@ua.edu.
